# Aberrant cerebral blood flow and functional connectivity in patients with vestibular migraine: a resting-state ASL and fMRI study

**DOI:** 10.1186/s10194-024-01792-5

**Published:** 2024-05-21

**Authors:** Zhengwei Chen, Yueji Liu, Cunxin Lin, Zhining Li, Junjun Shan, Zuowei Duan, Liangqun Rong, Xiue Wei, Lijie Xiao, Haiyan Liu

**Affiliations:** Department of Neurology, Second Affiliated Hospital of Xuzhou Medical University, No.32, Meijian Road, Xuzhou, Jiangsu 221006 China

**Keywords:** Vestibular migraine, Migraine, Arterial spin labeling, Cerebral blood flow, Functional magnetic resonance imaging, Functional connectivity

## Abstract

**Background:**

Prior neuroimaging studies on vestibular migraine (VM) have extensively certified the functional and structural alterations in multiple brain regions and networks. However, few studies have assessed the cerebral blood flow (CBF) in VM patients using arterial spin labeling (ASL). The present study aimed to investigate CBF and functional connectivity (FC) alterations in VM patients during interictal periods.

**Methods:**

We evaluated 52 VM patients and 46 healthy controls (HC) who received resting-state pseudo-continuous ASL and functional magnetic resonance imaging (fMRI) scanning. Comparisons of voxel-based CBF and seed-based FC were performed between the two groups. Brain regions showed significant group differences in CBF analyses were chosen as seeds in FC analyses. Additionally, the associations between abnormal imaging results and clinical features were explored.

**Results:**

Compared with HC, VM patients showed higher normalized CBF in the right precentral gyrus (PreCG), left postcentral gyrus (PostCG), left superior frontal gyrus and bilateral insular (*p* < 0.05, FDR corrected). Furthermore, VM patients exhibited increased FC between the right PreCG and areas of the left PostCG, left cuneus and right lingual gyrus (*p* < 0.05, FDR corrected). In addition, we observed decreased FC between the left insular and regions of the left thalamus and right anterior cingulate cortex, as well as increased FC between the left insular and right fusiform gyrus in VM patients (*p* < 0.05, FDR corrected). Moreover, these variations in brain perfusion and FC were significantly correlated with multiple clinical features including frequency of migraine symptoms, frequency of vestibular symptoms and disease duration of VM (all *p* < 0.05).

**Conclusions:**

Patients with VM during interictal period showed hyperperfusion and abnormal resting-state FC in brain regions potentially contributed to disrupted multi-sensory and autonomic processing, as well as impaired ocular motor control, pain modulation and emotional regulation. Our study provided novel insights into the complex neuropathology of VM from a CBF perspective.

## Background

Vestibular migraine (VM) is a central disorder characterized by recurrent vertigo and headache, accompanied by symptoms of nausea, vomiting, photophobia, phonophobia and imbalance [1]. VM is now considered the most common cause of episodic spontaneous vertigo and the second leading cause of vestibular syndromes ranking only second to benign paroxysmal positional vertigo [2, 3]. The lifetime prevalence of VM is 1-3% in general population [4, 5], and it is about five times more common in female when compared to male [2]. It has been reported that VM accounts for 9.0-11.9% of diseases in headache outpatient clinic, 6.0-25.1% of diseases in vertigo outpatient clinic, and 4.2–29.3% of diseases in otolaryngology outpatient clinic [6]. The recurrent vestibular and migraine symptoms seriously affect patients’ daily life, work and study, and also bring a heavy economic burden to the family and society.

To date, the pathological mechanisms of VM remain incompletely clear. Most theories concerning the pathogenesis of VM come from the researches related to migraine, including trigeminal neurovascular theory, cortical spreading depression, neurotransmitter abnormalities, ion channel defects, genetic factors, etc. [7, 8]. However, none of these theories can fully explain the clinical symptoms of VM.

Recently, neuroimaging studies that investigated brain structural and functional changes in VM patients have greatly improved people’s understanding of the neural basis of VM. By performing high resolution three-dimensional T1 weighted imaging (T1WI) on VM patients, voxel-based morphometry (VBM) studies observed that VM patients showed altered gray matter volume (GMV) in multi-sensory vestibular cortices [9–13]. However, the results of VBM studies vary greatly from one to another [9–13]. By performing diffusion tensor imaging (DTI), a tract based spatial statistic (TBSS) study found no microstructural changes in VM patients [14]. Using 18 F-fluorodeoxy glucose positron emission tomography (PET), Shin et al. reported increased metabolism in vestibulo-thalamo-cortical pathway and decreased metabolism in visual pathway in two VM patients during VM attack period [15].

Recent years, with high resolution and precision in time and space domain, functional magnetic resonance imaging (fMRI), a widely used neuroimaging technique to discover the brain functional alterations associated with neuropathology, has been increasingly adopted for researches to investigate the neural basis of VM. Two task-state fMRI studies using caloric vestibular stimulation or visual stimulation in patients with VM during attack-free period have suggested aberrant brain activation in thalamus, multi-modal association brain areas, occipital cortices and fronto-temporal regions [16, 17]. Different from task-state fMRI, resting-state fMRI examines changes of brain function in the basic state of the disease without any task or stimulation. Thus, resting-state fMRI is much easier to carry out and has been increasingly used in recent VM studies. Previous resting-state fMRI studies on VM mainly adopted analytic methods of amplitude of low-frequency fluctuation (ALFF), fractional ALFF (fALFF), regional homogeneity (ReHo), functional connectivity (FC), data-driven voxel-wise degree centrality (DC), independent component analysis (ICA), functional network connectivity (FNC), dynamic ALFF and ReHo, and dynamic FNC, etc [18–27]. . For example, Li et al. found increased ALFF values in the right temporal lobe and increased ReHo values in the right superior, middle, and inferior temporal gyrus in 17 definite VM patients compared with healthy controls (HC) [18]. Our previous work substantiated altered thalamo‑cortical FC and aberrant dynamic FNC between sensory networks and networks related to cognitive, emotional and attention implementation [20, 21]. Recently, a dynamic ALFF and ReHo study revealed altered temporal dynamics and concordance of regional intrinsic brain activity in the motor cortex, cerebellum, occipital and temporoparietal cortex in 57 VM patients during the interictal period when comparing to 88 HC [26].

It is apparent that neuroimaging studies using techniques of high-resolution T1WI, 18 F-fluorodeoxy glucose PET, task-state fMRI and resting-state fMRI to explore brain alterations in patients with VM have shown that multiple brain regions and networks involved in the pathological mechanism of VM, both structurally and functionally [9–27]. Undeniably, these neuroimaging studies extensively contributed to elucidate the complex mechanisms underpinning the pathophysiology of VM. However, the consistency and reproducibility of these literatures are often poor. This potentially has something to do with the relatively small sample size, clinical heterogeneity of subjects, different imaging devices and scanning parameters, and diverse imaging analytic methods in previous researches. In any case, from a neuroimaging perspective, there is no specific theoretical model for the pathological mechanism of VM until now.

According to the trigeminal neurovascular theory for migraine, using arterial spin labeling (ASL) technique, previous studies have reported the cerebral blood flow (CBF) alterations in migraine patients (both with and without aura) during attacks, and even during interictal periods [28–30]. ASL is a non-contrast MRI technique that enables the measurement of brain perfusion using magnetically labeled arterial blood water as an endogenous free diffusion tracer [31]. In contrast to other perfusion imaging techniques (for example, single photonemission computed tomography (SPECT), PET, CT perfusion and dynamic susceptibility contrast (DSC)), ASL has the advantages in terms of high reliability and repeatability, absolute quantification, avoidance of intravenous contrast administration (noninvasive) and superior spatial and temporal resolution [32]. To date, few studies have employed ASL imaging technique to evaluate the brain perfusion of patients with VM. It is currently not clear whether patients with VM showed abnormal CBF, nor do we know whether the brain regions with abnormal CBF in VM patients also exhibit aberrant resting-state FC.

Therefore, this study aimed to measure the pattern of CBF in patients with VM during interictal period using ASL. In addition, we explored whether the aberrant CBF brain regions of VM patients showed changes in resting-state FC using fMRI. Finally, we aimed to investigate the relationships between alterations in neuroimaging results (CBF and FC) and clinical characteristics of the patients. We assumed that the pattern of CBF and FC in patients with VM were different compared to those in HC. We also supposed that these alterations potentially correlated with certain clinical characteristics in the clinical practice.

## Methods

### Subjects and clinical assessment

Based on the diagnostic criteria for VM proposed by the International Bar´any Society in 2012 [33] and the International Classification of Headache Disorders (ICHD-3) in 2018 [34], we included sixty patients with VM from the Department of Neurology of the Second Affiliated Hospital of Xuzhou Medical University between January 2020 and August 2023. The VM was consistency diagnosed by two experienced vertigo specialists who have been engaged in clinical and research work on vertigo and VM for more than ten years. All patients were right-handed. All patients underwent routine physical examination of the nervous system. No patient showed positive neurological signs. To rule out peripheral vestibular diseases, all patients were evaluated by videonystagmograph (VNG), video head impulse test (vHIT) and vestibular evoked myogenic potentials (VEMPs). No patient showed obvious hearing impairment during audiometry tests. Patients with other neurological, neuro-otological, mental disorders, or systemic diseases were excluded. Patients with alcohol or drug abuse were excluded. Patients with a history of migraine with aura were excluded. All patients were scanned during interictal periods. Patients with symptoms of vertigo or migraine occurred 3 days before or after the fMRI and ASL scanning were excluded. All VM patients we included were not under regular preventive and curative medication within 3 days before fMRI and ASL evaluation. Demographic and clinical data were collected on all patients, including age, gender, educational level, duration of migraine, duration of VM, frequency of vestibular and migraine symptoms, Visual Analog Scale (VAS) for vertigo and headache (0, no pain or vertigo; 10, worst pain or vertigo), Montreal Cognitive Assessment (MoCA), Hamilton Anxiety scale (HAMA), Hamilton Depression scale (HAMD), Headache Impact Test-6 (HIT-6) and Dizziness Handicap Inventory (DHI).

The HC group was consisted of 50 age-, gender-, and education-matched volunteers. All of them were right-handed. None of them had a history of vertigo, migraine, other types of primary headache, and chronic pain. HC with a history of neurological, neuro-otological, psychic and systemic disorders were excluded. Volunteers with alcohol or drug abuse were excluded. All healthy subjects also received assessments of MoCA, HAMA and HAMD to excluded potential cognitive and psychological disorders. In addition, demographic information of age, gender and year of education were recorded. In order to rule out the influence of potential abnormal brain structure, lesions, intracranial artery stenosis and moderate to severe white matter hyperintensities on the CBF and FC, we performed conventional MRI for all patients and healthy volunteers, including scanning sequences of T1WI, T2 weight imaging (T2WI), diffusion weighted imaging (DWI), T2-FLAIR (fluid attenuated inversion recovery) and magnetic resonance angiography (MRA). They were further scanned for ASL and fMRI. This study was approved by the ethics committee of the Second Affiliated Hospital of Xuzhou Medical University and followed by the Declaration of Helsinki. Written informed consents were signed by all subjects before joining the study.

### MRI data acquisition

We acquired MRI data using a 3.0-Tesla MR scanner (Discovery MR750, GE Medical Systems, Milwaukee, WI, USA) with an 8-channel phased array head coil. Participants were required to keep their eyes closed, hold still, keep their minds relaxed, and stay awake without think of anything in particular during the scanning. According to the labeling scheme, ASL technique can be divided into pulsed ASL, continuous ASL and pseudo-continuous ASL (pc-ASL). In these three categories, the pc-ASL holds a more robust assessment of CBF, and provides both higher signal noise ratio (SNR) and labeling efficiency compared with pulsed ASL and continuous ASL respectively. Thus, we adopted a three-dimension pc-ASL with a fast spin-echo acquisition and background suppression [35]. The scanning parameters for pc-ASL were as follows: repetition time (TR) = 4886ms, echo time (TE) = 10.5ms, post-label delay = 2025ms, label duration = 1500 ms, flip angle (FA) = 111°, field of view (FOV) = 240 mm×240 mm, number of slices = 40, and number of excitation = 3. The resting-state fMRI data were obtained using a fast field echo-planar imaging (EPI) sequence with the following parameters: TR/TE = 2000/30ms, FA = 90°, FOV = 200 × 200 mm, thickness/gap = 3.6/0 mm, matrix = 64 × 64, and time points = 210. Three-dimension T1WI were collected using a brain volume (BRAVO) sequence (TR/TE = 2500/3.5ms, FA = 8°, FOV = 256 mm×256 mm, matrix = 256 × 256, thickness/gap = 1/0 mm, and number of slices = 156).

### Cerebral blood flow calculation

We used FuncTool software package (version 4.6, GE Medical Systems, Milwaukee, WI, USA) to generate the CBF images [36]. After format conversion (DICOM to NIFTI), the generated CBF maps were preprocessed using the Statistical Parameter Mapping (SPM; version 12; https://www.fil.ion.ucl.ac.uk/spm/software/spm12/) software package working on the platform of MATLAB (version 2023a, MathWorks, Inc., Natick, MA, USA). After the image quality check, the CBF images were normalized to the Montreal Neurological Institute (MNI) standard space with the following procedures: (a) we coregistered the subjects’ T1WI with the CBF images; (b) the coregistered T1WI were segmented and normalized to the MNI standard space; (c) the individual CBF image was brought into the MNI standard space and was resliced into a voxel size of 2 × 2 × 2 mm^3^. Then, we performed a quality check on the normalized CBF images for each subject. We removed the non-brain tissues from each co-registered CBF map. Affected by the efficiency of pc-ASL and individual haemodynamics, the CBF value of pc-ASL varies greatly among individuals. Thus, standardization was performed by dividing the CBF per voxel by the average CBF of the whole brain (Mean Division). The step of standardization was calculated using Data Processing and Analysis of Brain Imaging (DPABI, version 8.1, http://rfmri.org/dpabi) software. Finally, the standardized CBF maps were smoothed with an isotropic Gaussian kernel set at 8-mm full-width at half maximum.

### Functional MRI data preprocessing

Based on MATLAB platform, the resting-state fMRI data were preprocessed using SPM12 and the CONN software package (version 18b, http://www.nitrc.org/projects/ conn). The original DICOM format functional images and T1 weighted images were converted into NIFTI format using the Mricron toolbox (https://people.cas.sc.edu/rorden/mricron/). The first 10 time points were removed. The remaining 200 time points were preprocessed with the following steps: (a) functional slice-timing correction; (b) functional realignment (subject motion estimation and correction); (c) functional outlier detection (motion threshold: 3 mm; global signal threshold: z = 9); (d) to reduce the inconsistency of structural center caused by manual positioning by MR technicians during each scan, the structural center was set to coordinates of (0, 0, 0); (e) functional indirect segmentation and normalization (DARTEL); (f) functional smoothing (Gaussian kernel of 6 mm full-width at half maximum).

### Seedbased functional connectivity calculation

For the preprocessed functional images, to reduce low frequency drift and high frequency noise, we performed the steps of removal of linear trends and band-pass filtering (0.01–0.08 Hz). Next, removal of nuisance covariates were performed to minimize the influences of non-neuronal signals, including cerebrospinal fluid, white matter, global mean signals and head motion parameters. We adopted a seed-based FC method to calculate the FC between seeds and the rest of the brain regions. Five brain areas that exhibited significant group differences in normalized CBF were selected as seeds. The five seeds and their coordinates information were as follows: seed 1: right precentral gyrus (− 3, -6, 75); seed 2: left postcentral gyrus (-15, -27, 78); seed 3: left superior frontal gyrus (− 6, 42, 42); seed 4: left insular (-35, − 5, 2); seed 5: right insular (39, 2, 2). The seed region was defined as a sphere with a radius of 5 mm with the above five spatial coordinates as the center point. For the above five seeds, the mean time series were extracted from the previously processed functional images. Afterwards, Pearson correlation coefficients (r) between each seed and the rest of the brain voxels were calculated and converted to z-scores by Fisher’s r-to-z transformation.

### Statistical analyses

Demographic data and clinical features of the subjects were analyzed using Statistical Package for the Social Sciences (SPSS) for Windows (version 22.0, SPSS Institute Inc., Chicago, IL, USA). The statistical significance level was set at *p* < 0.05. Two-sample t-test was used to calculate the group differences in continuous parametric variables (age, educational level, scores of HAMA, HADM and MoCA). Chi-square test was adopted for the analysis of categorical variables (gender).

Between-group differences in normalized CBF and FC were calculated using two-sample t-test in SPM12 software package and the CONN toolbox respectively, with age, sex, educational level, scores of HAMA, HADM and MoCA as nuisance covariates. Significant threshold was set a voxel-level *p* < 0.05 with false discovery rate (FDR) correction for analyses of CBF and FC.

For brain regions showing significant differences in normalized CBF and FC between the two groups, the mean z-values were calculated within each patient with VM. Then, using SPSS software, we performed Pearson’s partial correlation analysis to calculate the relationships between z-values and patients’ clinical characteristics (including duration of VM, duration of migraine, frequency of vestibular/migraine symptoms, scores of vertigo/headache VAS, scores of HIT-6 and DHI), controlling for age, sex, educational years, scores of MoCA, HAMA and HAMD. A *p* < 0.05 was considered statistically significant.

## Results

### Demographic information and clinical characteristics of all subjects

Three patients were excluded because of VM attack. Two VM patients and one HC were excluded on account of poor normalization during CBF calculation. Three patients with VM and three HC were excluded due to large head motion and poor normalization during fMRI data processing. Finally, a total of 52 patients and 46 HC were included in the statistical analysis. Demographic and clinical data of the 52 patients and 46 HC were summarized in Table 1. There was no significant difference in terms of age, gender, educational level, scores of MoCA, HAMA and HAMD between patients with VM and HC (all *p* > 0.05). All VM patients had a migraine history without aura. 35 patients (67.3%) had a family history of migraine and 10 patients (19.2%) had a family history of VM. During the VM episode, all patients suffered from vertigo, including 28 (53.8%) patients of spontaneous vertigo, 11 (21.2%) patients of visually induced vertigo, and 13 (25%) patients of head-motion-induced vertigo. Headache, nausea, vomiting, photophobia and phonophobia occurred in 30 (57.7%), 31 (59.6%), 16 (30.8), 36 (69.2%) and 38 (73.1%) of all the 52 VM patients respectively during VM attacks.

**Table 1:** Demographic data and clinical features of the subjects

	VM(n = 52)	HC(n = 46)	*p*-value
Age (years)	37.44 ± 6.85	39.35 ± 6.69	0.168
Sex (female/male)	36/16	27/19	0.277
Education levels (years)	15.29 ± 3.01	16.04 ± 3.37	0.244
MoCA scores	27.58 ± 1.60	28.07 ± 1.54	0.128
HAMA scores	11.44 ± 4.38	10.07 ± 3.97	0.108
HAMD scores	9.19 ± 3.82	7.80 ± 4.38	0.097
Migraine duration (months)	139.77 ± 95.98	--	--
VM duration (months)	80.54 ± 34.10	--	--
Frequency of vestibular symptoms (d/m)	8.81 ± 4.12	--	--
Frequency of migraine symptoms (d/m)	6.48 ± 3.11	--	--
Vertigo VAS scores	6.87 ± 1.66	--	--
Headache VAS scores	4.77 ± 2.49	--	--
HIT-6 scores	56.06 ± 11.33	--	--
DHI scores	61.65 ± 19.62	--	--

*Abbreviations* VM, Vestibular migraine; HC, Healthy controls; MoCA, Montreal Cognitive Assessment; HAMA, Hamilton Anxiety Scale; HAMD, Hamilton Depression Scale; d/m, days per month; VAS, Visual analogue scale (0–10); HIT-6, Headache Impact Test-6; DHI, Dizziness Handicap Inventory

### Altered normalized CBF in patients with VM

**Fig. 1:**
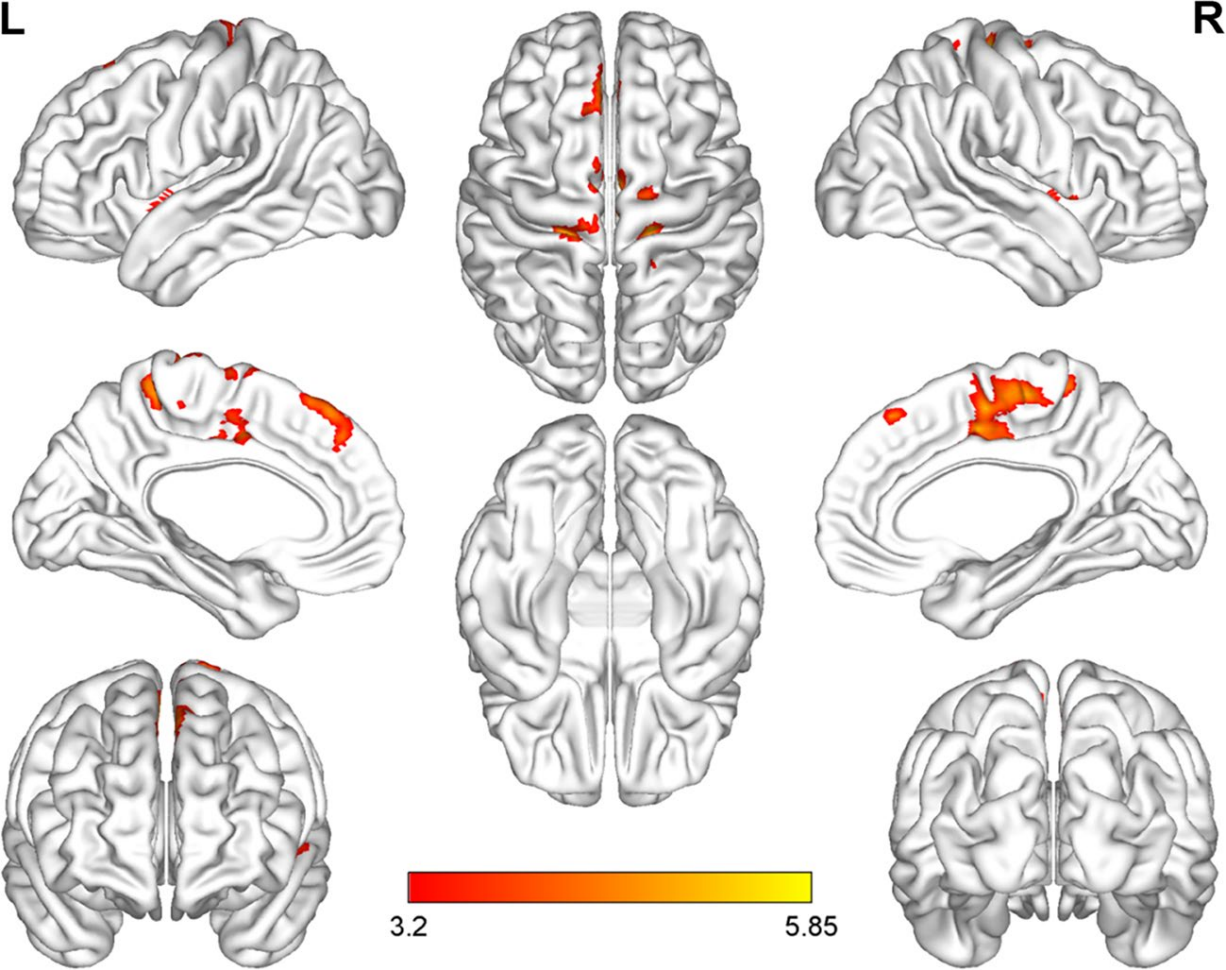
Brain regions with significant differences in normalized cerebral blood flow between patients with vestibular migraine and healthy controls (*p* < 0.05, FDR corrected). FDR, False discovery rate; L, Left; R, Right

**Fig. 2:**
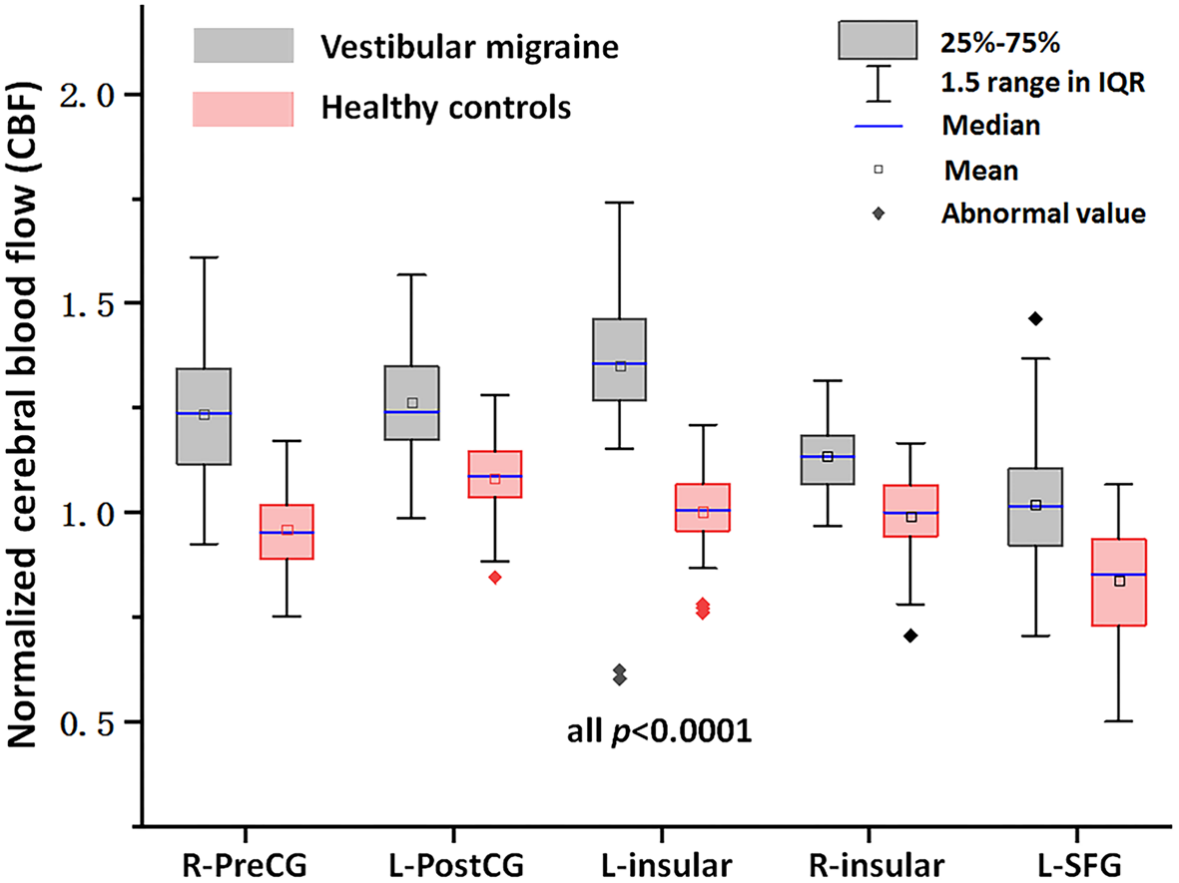
The normalized CBF values of patients with vestibular migraine and healthy controls (all *p* < 0.0001). IQR, Interquartile range; R-PreCG, Right precentral gyrus; L-PostCG, Left postcentral gyrus; L-SFG, Left superior frontal gyrus

The normalized CBF differences between patients with VM and HC were displayed in Table 2; Figs. 1 and 2. Compared with HC, patients with VM showed higher normalized CBF, primarily in brain regions of right precentral gyrus (PreCG), left postcentral gyrus (PostCG), left superior frontal gyrus (SFG) and bilateral insular (*p* < 0.05, FDR corrected).

**Table 2:** Brain regions with significant differences in CBF between patients with VM and HC

Brain regions	Cluster size (Voxels)	Peak MNI coordinates x, y, z	Peak t-value	AAL	BA
R-PreCG	533	-3 -6 75	5.8491	Supp_Motor_Area_R	6
L-PostCG	258	-15 -27 78	5.7958	Postcentral_L	4
L-SFG	149	-6 42 42	5.3153	Frontal_Sup_Medial_L	9
L-insular	135	-35 -5 2	4.7001	Insula_L	13
R-Insula	110	39 2 2	4.5162	Insula _R	13

Thresholds were set at *p* < 0.05 with False Discovery Rate (FDR) correction. CBF, Cerebral blood flow; VM, Vestibular migraine; HC, Healthy controls; MNI, Montreal neurological institute; AAL, Anatomical automatic labeling; BA, Brodmann area; L, Left; R, Right; PreCG, Precentral gyrus; PostCG, Postcentral gyrus; SFG, Superior frontal gyrus

### Altered FC in patients with VM

**Fig. 3:**
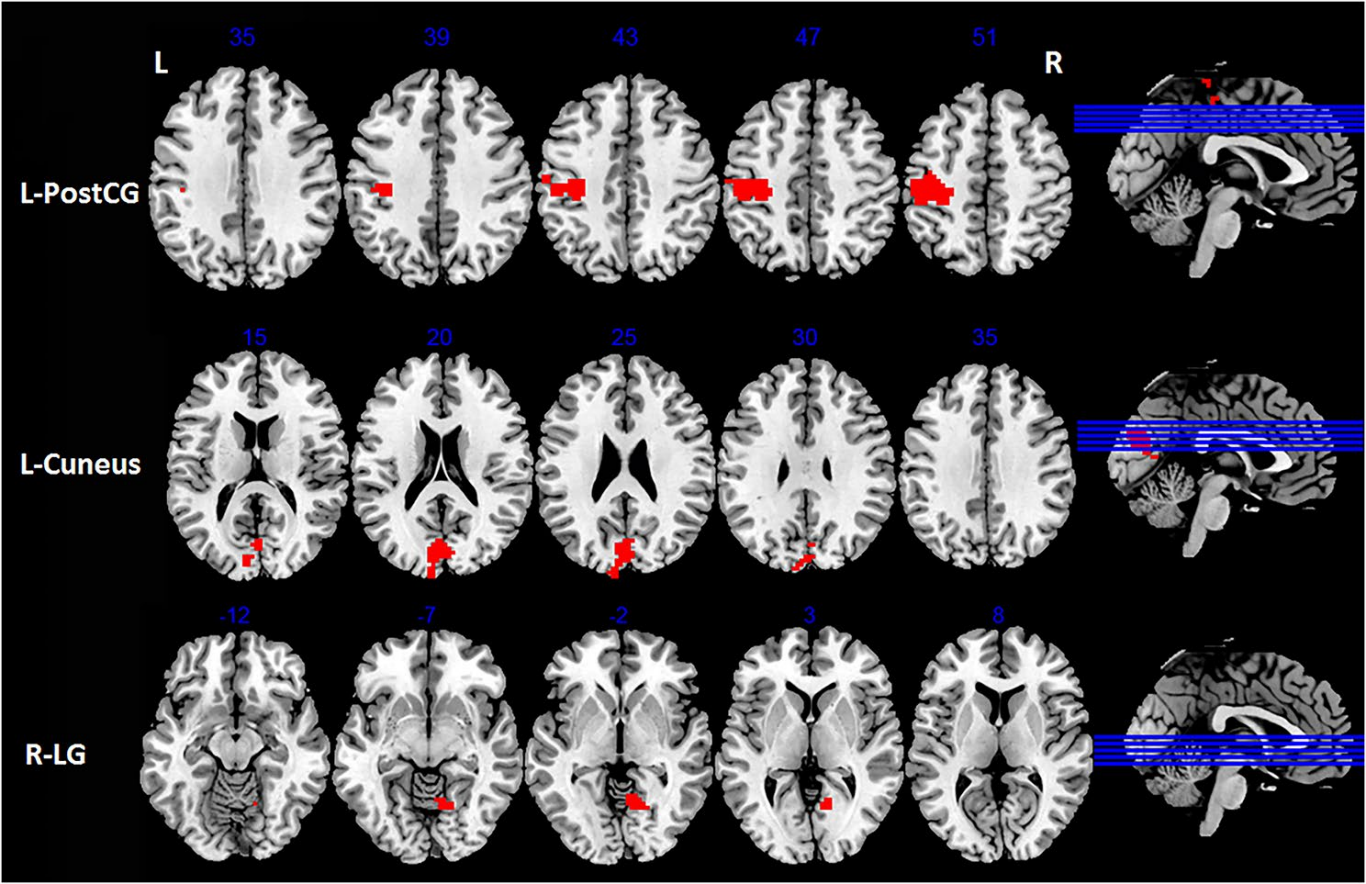
Brain regions showing significant differences in FC between VM patients and HC when the right precentral gyrus was chosen as a seed (*p* < 0.05, FDR corrected). Red areas indicate increased FC. FC, Functional connectivity; VM, Vestibular migraine; HC, healthy controls; FDR, False discovery rate; L, Left; R, Right. L-PostCG, Left postcentral gyrus; R-LG, Right lingual gyrus

**Fig. 4:**
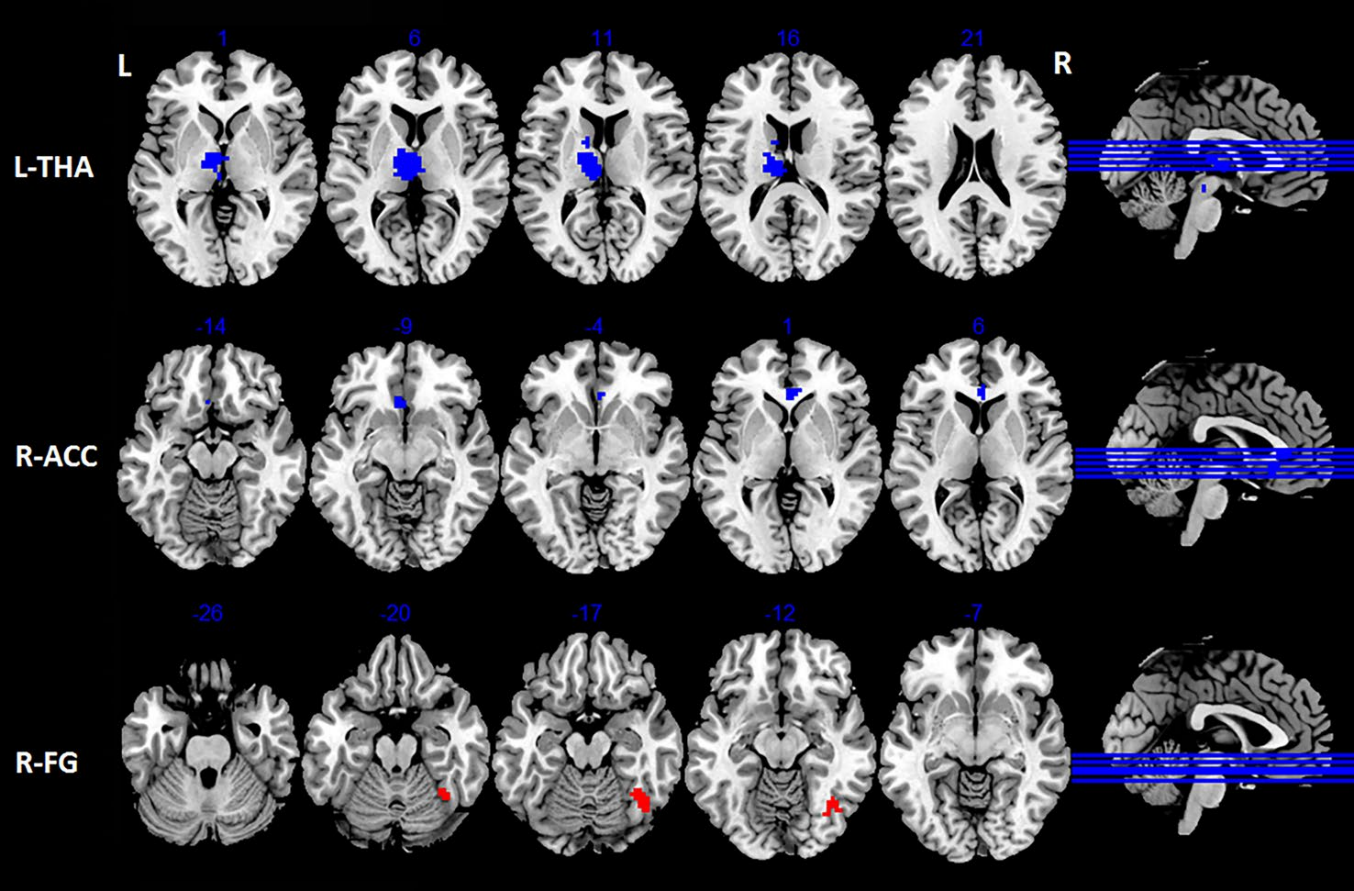
Brain regions showing significant differences in FC between VM patients and HC when the left insular was selected as a seed (*p* < 0.05, FDR corrected). Blue regions denote decreased FC, red areas indicate increased FC. FC, Functional connectivity; VM, Vestibular migraine; HC, healthy controls; FDR, False discovery rate; L, Left; R, Right. L-THA, Left thalamus; R-ACC, Right anterior cingulate cortex; R-FG, Right fusiform gyrus

**Fig. 5:**
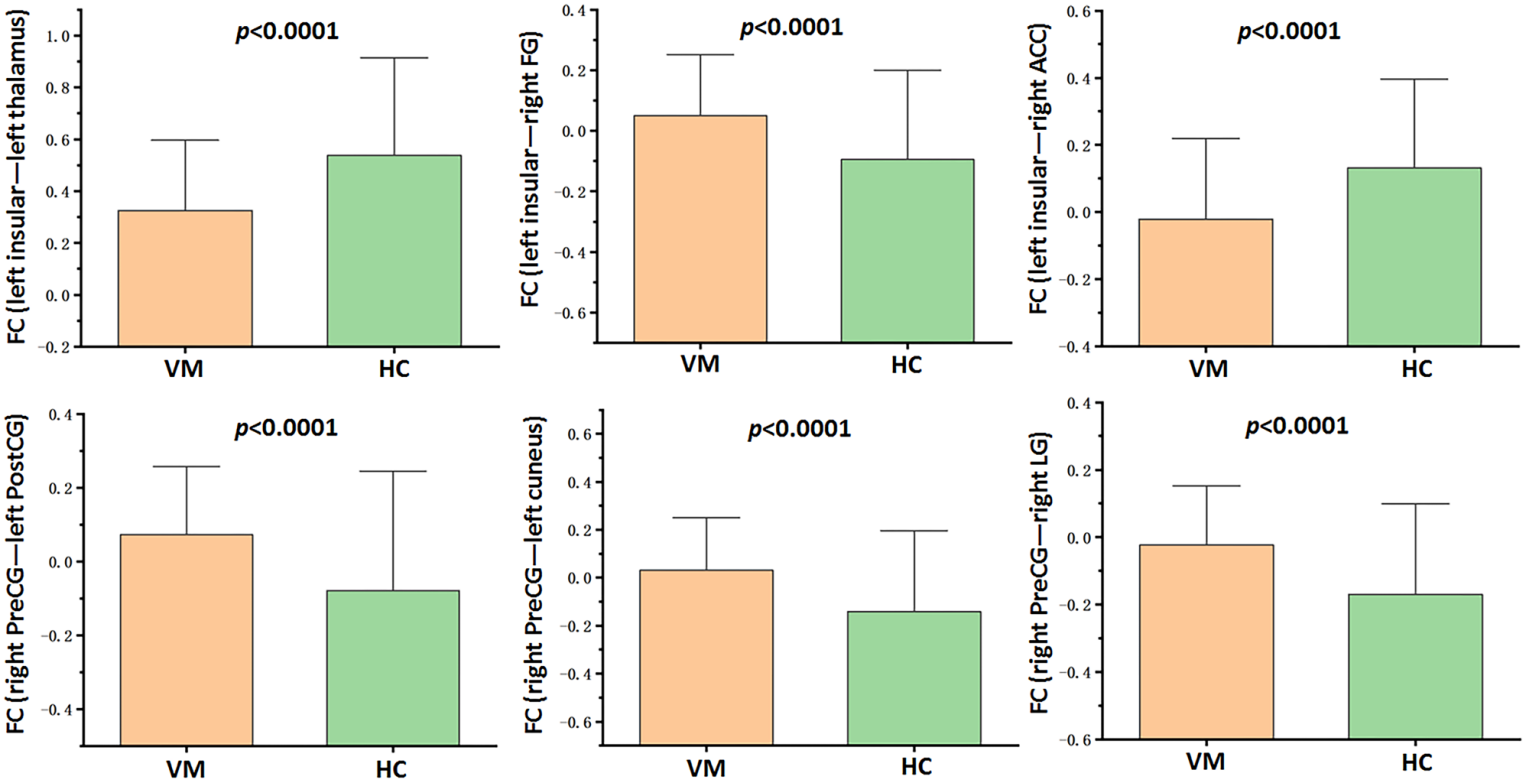
The FC values (z-values) of patients with vestibular migraine (VM) and healthy controls (HC) (all *p* < 0.0001). FC, Functional connectivity; FG, Fusiform gyrus; ACC, Anterior cingulate cortex; PostCG, Postcentral gyrus; LG, Lingual gyrus

Compared with HC, VM patients exhibited increased FC between right PreCG and areas of left PostCG, right lingual gyrus (LG) and left cuneus (*p* < 0.05, FDR corrected; Table 3; Figs. 3 and 5). In addition, we observed increased FC between left insular and right fusiform gyrus (FG) in VM patients (*p* < 0.05, FDR corrected; Table 4; Figs. 4 and 5). Furthermore, patients with VM showed decreased FC between left insular and brain regions of right anterior cingulate cortex (ACC) and left thalamus (*p* < 0.05, FDR corrected; Table 4; Figs. 4 and 5).

**Table 3:** Altered FC of right precentral gyrus in VM patients compared with HC

Brain regions	Cluster size (Voxels)	Peak MNI coordinates x, y, z	Peak t-value	AAL	BA
L-PostCG	560	-45 -24 54	5.7997	Postcentral_L	1
L-Cuneus	157	-6 -84 18	4.7449	Cuneus_L	13
R-LG	60	12–60 0	6.1209	Lingual_R	24

Significant threshold was set at *p* < 0.05 (FDR corrected). FC, Functional connectivity; VM, Vestibular migraine; HC, healthy controls; MNI, Montreal neurological institute; AAL, Anatomical automatic labeling; BA, Brodmann area; L, Left; R, Right; PostCG, Postcentral gyrus; LG, Lingual gyrus

**Table 4:** Altered FC of left insular in VM patients compared with HC

Brain regions	Cluster size (Voxels)	Peak MNI coordinates x, y, z	Peak t-value	AAL	BA
L-thalamus	230	-5 -15 7	-6.1124	Thalamus_L	50
R-ACC	66	2 32 2	-5.2518	Cingulum_Ant_R	24
R-FG	57	39–57 -18	4.4562	Fusiform_R	37

Significant threshold was set at *p* < 0.05 (FDR corrected). FC, Functional connectivity; VM, Vestibular migraine; HC, healthy controls; MNI, Montreal neurological institute; AAL, Anatomical automatic labeling; BA, Brodmann area; L, Left; R, Right; ACC, Anterior cingulate cortex; FG, Fusiform gyrus

### Correlations between the imaging data and clinical features in patients with VM

**Fig. 6:**
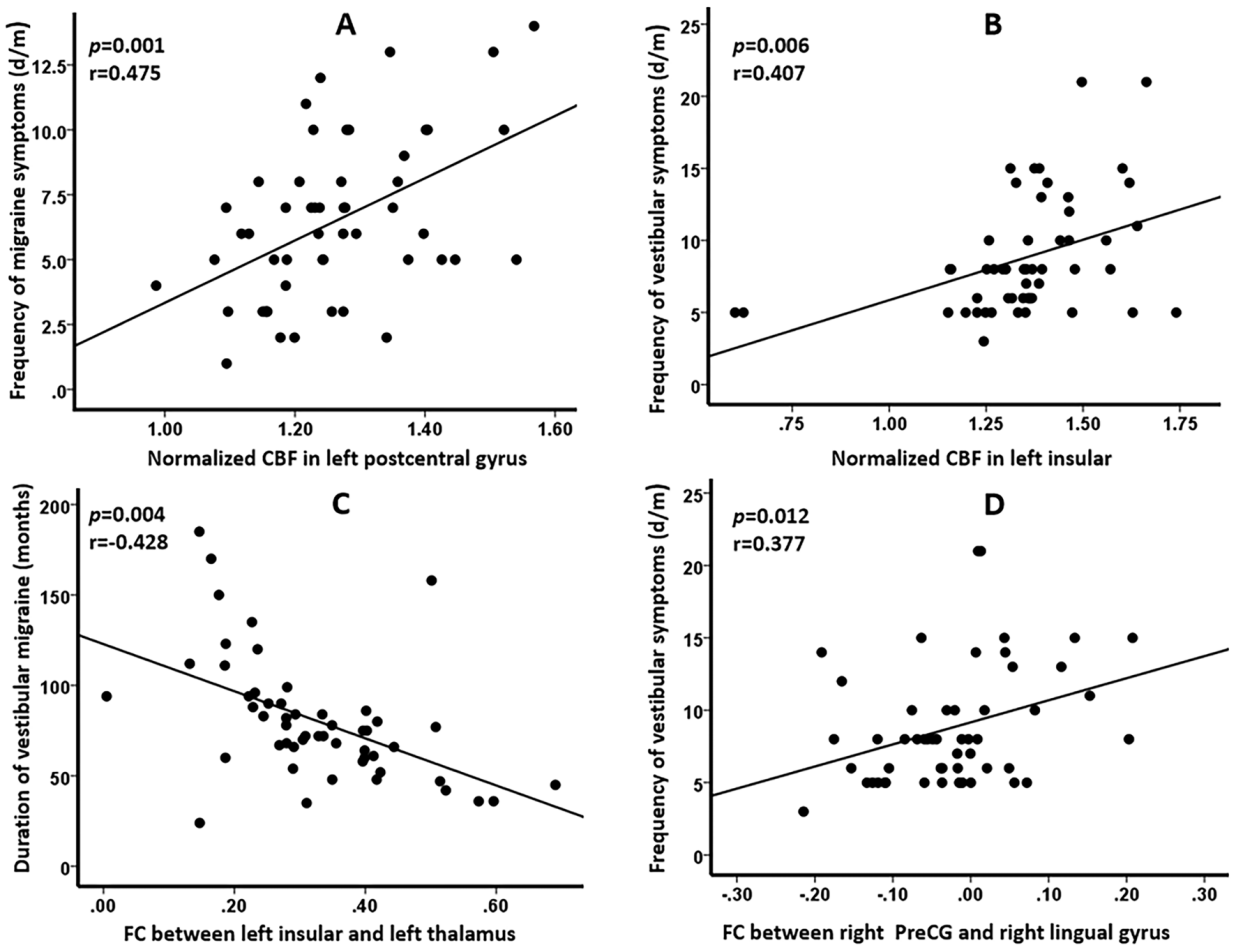
The significant correlations between neuroimaging changes and clinical data in patients with vestibular migraine. (**A**) Normalized cerebral blood flow (CBF) in left postcentral gyrus was positively correlated with the frequency of migraine symptoms (*p* = 0.001, *r* = 0.475); (**B**) Normalized CBF in left insular was positively correlated with the frequency of vestibular symptoms (*p* = 0.006, *r* = 0.407); (**C**) Functional connectivity (FC) between left insular and left thalamus was negatively correlated with the duration of vestibular migraine (*p* = 0.011, *r*= -0.352); (**D**) FC between right precentral gyrus (PreCG) and right lingual gyrus was positively correlated with the frequency of vestibular symptoms (*p* = 0.012, *r* = 0.377)

The significant correlations between the imaging changes and the clinical features in patients with VM were displayed in Fig. 6. The normalized CBF in left PostCG was positively correlated with the frequency of migraine symptoms (*p* = 0.001, *r* = 0.475), and the normalized CBF in left insular was positively correlated with the frequency of vestibular symptoms (*p* = 0.006, *r* = 0.407). In addition, the FC between left insular and left thalamus was negatively correlated with the duration of vestibular migraine (*p* = 0.011, *r*= -0.352), and the FC between right PreCG and right LG was positively correlated with the frequency of vestibular symptoms (*p* = 0.012, *r* = 0.377).

## Discussion

To our knowledge, this is the first resting-state pc-ASL combined with fMRI study designed to investigate alterations of CBF and FC in patients with VM during interictal periods. Our study revealed higher normalized CBF and abnormal FC in patients with VM when comparing to HC. In addition, the aberrant brain perfusion and FC were correlated with certain clinical features of the patients in clinical practice.

### Altered normalized CBF in VM patients

As far as we know, no literature has reported the abnormal CBF in VM patients using ASL technique. We conducted a voxel-wise analysis to extract brain blood flow features quantificationally which significantly contribute to the pathophysiology of VM. We found higher normalized CBF in the right PreCG (right supplementary motor area (SMA) corresponding to anatomical automatic labeling (AAL)) in patients with VM. The PreCG is included in human primary motor cortex. The SMA and PreCG are core parts of the cortico-basal-thalamo-motor cortical system [37, 38]. It was reported that the PreCG/SMA was usually activated in response to painful heat stimuli [39]. The PreCG was also observed to be significantly activated during caloric or electrical vestibular stimulation [40, 41]. Previous neuroimaging studies have reported the functional and structural changes of PreCG in migraine patients compared to HC [42, 43]. In patients with VM, a recent dynamic ALFF and ReHo study observed increased regional intrinsic brain activity converged in the motor cortex, including the PreCG, SMA and paracentral lobule [26]. Their findings of increased intrinsic brain activity in motor cortex were similar with our results that discovered increased CBF in the PreCG. Our results were also partially in accordance with previous neuroimaging studies that observed altered resting-state FC and GMV in SMA in patients with VM [12, 20, 44]. Patients with VM during an episode often experience motion-induced vertigo and imbalance. The increased CBF in PreCG/SMA in our study potentially associated with the impairments in motor control and postural stability.

The PostCG is a crucial adjective component of human primary somatosensory cortex and plays an important role in the trigemino-thalamo-cortical pathway as demonstrated by previous studies that explored the pathophysiology of migraine [45–47]. Although the functional and structural changes of PostCG in migraine patients have been extensively observed [47–50], it is regrettable that the functional and structural alterations of PostCG in VM patients have been rarely reported in the literatures [9–27]. This study found increased normalized CBF in the left PostCG in VM patients when comparing with HC, which resembled a recent ASL study that observed higher CBF levels in the bilateral PostCG in migraine patients with aura when comparing with migraine patients without aura and HC [51]. Furthermore, we found that the normalized CBF in left PostCG was positively correlated with the frequency of migraine symptoms in patients with VM. Therefore, we suggested that the increased brain perfusion in PostCG was associated with the deficits of trigemino-thalamo-cortical pathway in patients with VM, and was potentially related to balance disorders in VM patients as somatosensory system is an important factor in maintaining balance.

Research has discovered that, unlike other sensory system, the human vestibular areas consisted of various brain regions [52], including the SFG [53, 54], parieto-insular vestibular cortex (PIVC) [55, 56], thalamus [57], angular gyrus [53, 58], middle frontal gyrus [53, 54, 59], hippocampus [58], supramarginal gyrus [53], etc. The SFG within vestibular system was reported to be responsible for ocular motor control and was involved in the processing of nystagmus, as identified by neuroimaging studies during peripheral vestibular caloric and galvanic stimulation [53, 54]. In addition, a previous study confirmed that the SFG was involved in the integration of somatosensory and vestibular information [60]. Alterations of SFG in task-state functional activation, resting-state FC and GMV were previously demonstrated in patients with VM [12, 17, 44, 61]. The SFG was also suggested to be related to pain modulation [62]. Increased FC between the left thalamus and the left SFG in migraine patients was previously reported [63]. In addition, a study using ASL technique observed higher CBF levels in the bilateral SFG in migraine patients with aura in comparison to migraine patients without aura and HC [51]. However, another ASL study found decreased CBF in the left SFG in migraine patients with tinnitus [64]. The current study found higher CBF in the left SFG in VM patients. We suggested that the increased cerebral perfusion in SFG was associated with impaired central vestibular processing and pain modulation in VM patients.

Patients with VM also exhibited higher normalized CBF in the bilateral insular when compared with HC. It was reported that the PIVC was the core of the human vestibular areas and was considered to be the primary vestibular cortical region in humans [65]. The insular, a vital component of human PIVC, plays a crucial role in the vestibulo-thalamo-cortical pathway and is responsible for receiving vestibular information and signal processing [52, 55, 56]. Lesion studies have shown that damaged insula area affects the perception of verticality or causes vertigo [66, 67]. The insular cortex is also engaged in pain perception, coding, and regulation [68]. It receives pain input from the spino-thalamo-cortical pathway [68]. Furthermore, the insular cortex is also suggested to be a fundamental part of central autonomic network and mediates high-order autonomic control [69, 70]. Previous neuroimaging studies using 18 F-fluorodeoxy glucose PET, high resolution T1WI and resting-state fMRI have proved the brain metabolism, GMV and FC alterations in the insular cortices in patients with VM [9, 15, 20, 22]. The results of increased insular CBF in this study are similar to those of the above neuroimaging studies. In addition, it further confirmed from the perspective of CBF that the insula cortices might play an important role in the pathogenesis of VM. We also observed a significant positive correlation between the normalized CBF in left insular and the frequency of vestibular symptoms in VM patients. Therefore, we believed that recurrent vestibular and autonomic (nausea and vomiting) symptoms were potentially related to cerebral perfusion abnormalities in the insular regions.

### Altered resting-state FC in VM patients

Based on blood oxygen level-dependent (BOLD), the resting-state fMRI reflected inherent abnormality and revealed more fundamental brain functional alterations in patients with neurological and psychotic disorders [71–73]. Using resting-state fMRI technique, previous studies have reported the intrinsic abnormalities in brain functional activity in patients with VM, which involve many brain regions and networks [10, 18–27]. The FC analyses in the current study revealed significant increased FC between right PreCG and left PostCG in patients with VM, indicating enhanced functional interactions between motor and somatosensory networks (namely, within sensorimotor network). A previous VM study suggested that the impaired sensorimotor network was associated with a hypersensitivity state (photophobia/phonophobia), but the authors observed decreased FC within the sensorimotor network that is partly contrary to the results of the current study [19]. The cuneus and LG are the main components of primary visual network, which are responsible for visual information processing [74]. Thus, the increased FC between right PreCG and regions of left cuneus and LG suggested enhanced functional connections between motor and visual networks. Similar to our study, previous MRI studies on VM have reported the functional changes in the cuneus and LG [17, 24, 61]. Additionally, we found that the FC between right PreCG and right LG was positively correlated with the frequency of vestibular symptoms. The altered FC in motor, somatosensory and visual cortices potentially reflects the impairments in multi-sensory processing, which is most likely an adaptive change in response to recurrent vestibular symptoms.

The thalamus, a crucial adjective of trigemino-thalamo-cortical circuit, serves as a transfer station for pain conduction [75, 76]. The thalamus also plays an important role in central vestibular circuit as it receives peripheral vestibular input from the vestibular nuclei and transfers vestibular information to the central vestibular cortex [77]. The important role of thalamus in the pathophysiological mechanism of migraine patients has been confirmed by neuroimaging studies using fMRI [16, 75], diffusion tensor imaging [76] and ASL [51]. The present study observed decreased FC between the left thalamus and left insular. In addition, the FC between left insular and left thalamus was negatively correlated with the disease duration of VM. Our results indicated decreased vestibulo-thalamo-cortical pathway in VM patients. Our findings were in agreement with previous literatures that reported altered thalamus functional activation during ear irrigation with cold water [16], resting-state FC [20, 25] and ALFF values [25]. The ACC was considered a key region of emotional regulation, especially the rostral ACC [78, 79]. The present study showed decreased FC between the left insular and right ACC (right rostral ACC, to be exact), indicating impaired emotional regulation in patients with VM. We also observed increased FC between the left insular and the right FG. The FG is engaged in higher visual function and the processing of pain sensory [80, 81]. Similar to the results of our study, previous studies have demonstrated that FG was functionally and structurally altered in migraineurs compared to HC [42, 80]. Our results indicated disrupted integration among vestibular, visual, and pain sensory in patients with VM.

According to the neurovascular coupling hypothesis, brain areas with stronger resting-state FC tend to have higher spontaneous neuronal activity with greater metabolic demand, resulting in increased regional CBF [82, 83]. To support this hypothesis, it was reported that a higher degree of FC was related to an increase in glucose metabolism in healthy group [84]. In addition, previous seed-based FC studies have demonstrated the relations between CBF and FC during resting state [85, 86]. It is worth noting that not all CBF increased brain regions showed abnormal FC in the present study. Furthermore, a brain region (left insular) with increased CBF showed decreased FC in the current study (decreased FC between left insular and regions of left thalamus and right ACC). Therefore, we assumed that patients with VM potentially showed aberrant neurovascular coupling between resting-state CBF and FC. However, this prediction needs to be confirmed by relevant analytical methods in future studies.

### Limitations

Several potential limitations of this study need to be mentioned. First, although the sample size of the present study was relatively larger than most of the existing neuroimaging studies on VM, researches with larger number of VM patients are urgently needed in future to improve the generalization of the results. Second, the male to female composition ratio of VM patients in this study was 1:2.25. The potential influence of gender-related variations on resting-state CBF and FC changes could not be completely ruled out. Third, similar to most previous studies, the VM patients included in this study exhibited relatively large variation in disease duration and frequency of attacks, more tight range in the symptomatology should be adopted. Fourth, the correlation analysis in the present study was not corrected by multiple comparison correction (for example, the Bonferroni correction). Another potential limitation was the lack of a control group consisted of migraineurs without vestibular symptoms. Last but not least, this cross-sectional study design failed to make a definitive judgment on the causal relationship between aberrant CBF/FC and VM symptoms.

## Conclusions

In conclusion, patients with VM during interictal period showed higher normalized CBF in sensorimotor cortex, insular and SFG. Furthermore, the current study revealed altered resting-state FC in sensorimotor and visual cortices, insular, thalamus, rostral ACC and LG. These areas that showed hyperperfusion and abnormal FC potentially contributed to disrupted multi-sensory and autonomic processing, as well as impaired ocular motor control, pain modulation and emotional regulation in patients with VM during interictal period. Our study provided novel insights into the complex neuropathology of VM from a CBF perspective.

## Data Availability

Data that support the results of this study are available from the corresponding authors on reasonable request.

## Abbreviations

VM: Vestibular migraine
VBM: Voxel-based morphological
T1WI: T1 weighted imaging
GMV: Gray matter volume
PET: Positron emission tomography
fMRI: Functional magnetic resonance imaging
ALFF: Amplitude of low-frequency fluctuation
ReHo: Regional homogeneity
FC: Functional connectivity
FNC: Functional network connectivity
HC: Healthy controls
ASL: Arterial spin labeling
CBF: Cerebral blood flow
VAS: Visual analog scale
MoCA: Montreal cognitive assessment
HAMA: Hamilton anxiety scale
HAMD: Hamilton depression scale
HIT-6: Headache impact test-6
DHI: Dizziness handicap inventor
pc-ASL: Pseudo-continuous ASL
PreCG: Precentral gyrus
PostCG: Postcentral gyrus
SFG: Superior frontal gyrus
LG: Lingual gyrus
FG: Fusiform gyrus
ACC: Anterior cingulate cortex
SMA: Supplementary motor area
